# Exercise-induced muscle extracellular vesicles reprogram brain microglia to enhance amyloid clearance in Alzheimer’s disease

**DOI:** 10.20517/evcna.2026.73

**Published:** 2026-07-09

**Authors:** Toshihide Takeuchi, Yoshitaka Nagai

**Affiliations:** ^1^Life Science Research Institute, Kindai University, Osaka 590-0197, Japan.; ^2^Department of Neurology, Kindai University Faculty of Medicine, Osaka 590-0197, Japan.

## MAIN TEXT

Regular physical exercise is one of the most effective non-pharmacological strategies for preserving cognitive function and lowering the risk of Alzheimer’s disease (AD). Despite strong epidemiological and clinical evidence, the mechanisms linking physical activity to cognitive benefit remain unclear. Skeletal muscle is widely recognized as an endocrine organ that communicates with distant tissues via secreted factors. Among the mediators of this systemic crosstalk are extracellular vesicles (EVs), which carry proteins, nucleic acids, lipids, and other bioactive molecules that modulate recipient cell function. Muscle-derived EVs have been proposed to mediate communication between skeletal muscle and peripheral organs such as adipose tissue and the liver^[1]^. Physical exercise also alters the abundance and cargo composition of circulating EVs, thereby implicating EVs in systemic metabolic adaptation and inter-organ communication during exercise^[2]^. Recent studies further suggest that exercise-induced EVs can ameliorate AD-associated peripheral metabolic deficits^[3]^ and reduce brain amyloid pathology in animal models^[4]^. However, the mechanistic link between exercise-induced circulating EVs and amyloid clearance remains poorly defined.

In this context, Lin *et al.* provide experimental evidence for a muscle-brain EV signaling pathway that directly regulates the protective activity of microglia in AD^[5]^. AD is characterized by progressive cognitive decline, accompanied by amyloid-β (Aβ) deposition and neuroinflammation in the brain. Using a mouse model of AD (APP/PS1 mice), the authors show that a four-week swimming protocol significantly improves cognitive performance and reduces Aβ plaque burden in the cortex and hippocampus. These benefits are associated with marked changes in microglial state. Exercise induced disease-associated microglia (DAM), which is a microglial phenotype linked to enhanced phagocytic activity and Aβ clearance in early AD^[6]^. Notably, the depletion of microglia abolishes the cognitive benefits of exercise and the reduction in amyloid pathology, demonstrating that microglia are essential for the protective effects of exercise in AD.

A key advance of this study is the identification of skeletal muscle-derived EVs (SKM-EVs) as mediators linking physical exercise to microglial activation. The authors show that exercise increases EV biogenesis and secretion from skeletal muscle, both *in vivo* and in an *ex vivo* muscle contraction model. Using skeletal muscle-specific CD63-GFP reporter mice, they further demonstrate that muscle-derived EVs can reach the brain and are taken up by microglia. In addition, intravenous administration of exercise-induced SKM-EVs was found to recapitulate the beneficial effects of exercise on cognitive function, amyloid pathology, and microglial activation, whereas the inhibition of muscle EV secretion abolishes these effects. Taken together, these data provide strong evidence for an EV-mediated muscle-brain communication pathway that regulates AD-associated brain pathology.

This study provides further mechanistic insight by identifying miR-378a-3p as a key EV cargo in exercise-induced SKM-EVs. MicroRNA profiling demonstrated that miR-378a-3p was enriched in EVs released after exercise and was increased not only in skeletal muscle but also in the cortex and hippocampus. Direct delivery of this microRNA improved cognitive function and enhanced microglial phagocytosis, whereas blocking muscle miRNA processing or silencing miR-378a-3p abolished the beneficial effects of exercise. Mechanistically, miR-378a-3p appears to act by reprogramming microglial metabolism, as it targets p110α, the catalytic subunit of phosphoinositide-3-kinase (PI3K), and modulates the PI3K-AKT-FoxO1 signaling pathway, leading to enhanced lipid metabolism and ATP production in microglia. This metabolic shift supports the high energetic demands of microglial phagocytosis and promotes DAM activation. These findings identify a major signaling axis underlying the benefits of exercise in AD: skeletal muscle as the source, EVs as the carrier, miR-378a-3p as the cargo, microglia as the recipient, and amyloid clearance as the functional outcome.

The translational implications of these findings are also important. Many patients with AD may be unable to sustain the level of physical activity required to achieve meaningful benefit, particularly as the disease progresses. Lin *et al.* demonstrate that EVs derived from miR-378a-3p-overexpressing myotubes can mimic the effects of exercise, improve cognitive function, and reduce Aβ pathology in AD mice^[5]^. These findings suggest the potential for developing EV-based “exercise mimetics” as a therapeutic strategy, particularly for patients with limited mobility or those unable to sustain regular physical activity. In preclinical studies, EVs have shown the capacity to deliver therapeutic cargo to the brain with low apparent immunogenicity^[7]^, supporting their development as vehicles for neurological intervention. However, these findings are currently limited to mouse models, and several limitations remain, including challenges in translation to humans. The duration of action of the administered muscle-derived EVs has not yet been determined, and the optimal dosing interval for sustained benefit remains unclear. Although microglia were identified as a major recipient cell type, they may not be the sole targets in the brain; astrocytes, neurons, and vascular cells may also contribute to the response to exercise-induced EVs. In addition, the mechanisms underlying EV biogenesis and increased EV release from skeletal muscle during exercise are not yet fully understood. Whereas EV cargo is complex, it remains unclear whether components other than miR-378a-3p contribute to the observed benefits. Because microglial responses vary over the course of AD, the timing of intervention may be crucial. Future studies in humans will be essential to determine whether this EV-mediated muscle-brain communication pathway is conserved and therapeutically applicable.

In summary, Lin *et al.* identify a mechanistic link between exercise and its cognitive benefits in AD by demonstrating that SKM-EVs deliver miR-378a-3p to microglia, reprogramming their metabolism to enhance Aβ clearance and improve cognitive function^[5]^. This study provides a compelling framework for understanding how EVs mediate the systemic benefits of exercise through inter-organ communication, while also highlighting their therapeutic potential in AD.

## DECLARATIONS

### Copyright

© The Author(s) 2026.

## References

[1] (2021). Vechetti IJ Jr, Valentino T, Mobley CB, McCarthy JJ. The role of extracellular vesicles in skeletal muscle and systematic adaptation to exercise. J Physiol.

[2] Whitham M, Parker BL, Friedrichsen M (2018). Extracellular vesicles provide a means for tissue crosstalk during exercise. Cell Metab.

[3] Fuller OK, McLennan ED, Egan CL (2025). Extracellular vesicles contribute to the beneficial effects of exercise training in APP/PS1 mice. iScience.

[4] Takeda A, Takeuchi T, Minakawa EN, Tanaka N, Mochizuki H, Nagai Y (2026). Blood extracellular vesicles contribute to the exercise-mediated suppression of brain Aβ pathology in the App^NL-G-F^ knockin mouse model of Alzheimer’s disease. Neurochem Int.

[5] Lin J, Shao X, Shi T (2026). Exercise alleviates cognitive dysfunction in Alzheimer’s disease mice via skeletal muscle-derived extracellular vesicles that enhance plaque clearance by microglia. Nat Aging.

[6] Keren-Shaul H, Spinrad A, Weiner A (2017). A unique microglia type associated with restricting development of Alzheimer’s disease. Cell.

[7] Alvarez-Erviti L, Seow Y, Yin H, Betts C, Lakhal S, Wood MJ (2011). Delivery of siRNA to the mouse brain by systemic injection of targeted exosomes. Nat Biotechnol.

